# Mental health of college sex workers

**DOI:** 10.3389/fpubh.2026.1800468

**Published:** 2026-04-14

**Authors:** Stacie Lefeavers, Abigail H. Conley, Karen Chartier, Spit for Science Work Group, Karen Chartier, Ananda Amstadter, Danielle M. Dick, Emily Lilley, Renolda Gelzinis, Anne Morris, Katie Bountress, Amy E. Adkins, Nathaniel Thomas, Zoe Neale, Kimberly Pedersen, Thomas Bannard, Seung B. Cho, Kimberly Pedersen, Amy E. Adkins, Peter Barr, Holly Byers, Erin C. Berenz, Erin Caraway, Seung B. Cho, James S. Clifford, Megan Cooke, Elizabeth Do, Alexis C. Edwards, Neeru Goyal, Laura M. Hack, Lisa J. Halberstadt, Sage Hawn, Sally Kuo, Emily Lasko, Jennifer Lent, Mackenzie Lind, Elizabeth Long, Alexandra Martelli, Jacquelyn L. Meyers, Kerry Mitchell, Ashlee Moore, Arden Moscati, Aashir Nasim, Zoe Neale, Jill Opalesky, Cassie Overstreet, A. Christian Pais, Tarah Raldiris, Jessica Salvatore, Jeanne Savage, Rebecca Smith, David Sosnowski, Jinni Su, Nathaniel Thomas, Chloe Walker, Marcie Walsh, Teresa Willoughby, Madison Woodroof, Jia Yan

**Affiliations:** 1 Virginia Commonwealth University, Social and Behavioral Science, School of Public Health, Richmond, VA, United States; 2 Department of Counseling and Special Education, Virginia Commonwealth University, Richmond, VA, United States; 3 Institute for Research on Behavioral and Emotional Health & Spit for Science Registry, Virginia Commonwealth University, Richmond, VA, United States

**Keywords:** ACES, anxiety, college students, depression, sex work, sexual assault

## Abstract

This study explored the impact of developmental and recent traumas on PTSD, anxiety, and depression among a sample of university students (*N* = 410), including students who engaged in sex work during college. Using linear and hierarchical regressions, the study examined whether gender-based violence and potentially exploitative activities in college, specifically sexual assault (SA), sex work, sex trafficking, and intimate partner violence (IPV) contributed to mental health outcomes when controlling for childhood history. Post-traumatic stress (*n* = 153) was primarily predicted by more recent SA. In contrast, anxiety symptoms (*n* = 410) were more closely tied to child sex abuse (CSA). In a hierarchical model for depression, childhood peer sexual abuse (*p* = 0.021), college-era SA (*p* = 0.032), and sex work involvement (*p* = 0.042) emerged as significant independent predictors. The results underscore the need for holistic, trauma-informed clinical interventions on campuses that address both historical and current stressors, including the specific psychological needs of students involved in sex work.

## Introduction

In the United States, financial stressors associated with college education can significantly increase students' vulnerability to exploitation. Some students seek to earn extra income by participating in online sex work, while others participate in arrangement relationships that may or may not involve a sexual relationship (37). Still, others find themselves working in the commercial sex industry not by choice—they have been coerced or threatened to do so. Although willing sex work is not the same as human trafficking, which requires the presence of force, fraud, and/or coercion, situations involving the exchange of sexual services frequently involve power imbalances that can lead to exploitation. Collecting prevalence data on internet-facilitated sex work and arrangement relationships can inform higher education efforts to understand how to best support these students.

“Sex work” or “sex trading” can encompass a range of activities that take place online or in person. For instance, “camming” refers to creating adult content on a livestream through a webcam. Cam models can join an established web platform or build their own in order to gain subscribers and process payments. One of the most well-known platforms, OnlyFans, saw a sharp rise in popularity during the COVID-19 pandemic as workers faced layoffs and furloughs during lockdown. At the same time, traditional venues for recreation were shut down, leading people to turn to new sources of entertainment. In December 2020 the platform grew to 85 million users, up from 7.5 million in November 2019 (1). Despite the popularity of the platform, OnlyFans users may go months without payments due to the challenge of attracting subscribers and the oversaturation of the space. Instead of trying to build a business with followers, some students who engage in sex work look for quick transactions like selling photos, and can typically find buyers on dating apps (2). Another short-term transaction that students utilize is selling used clothing items that are packaged and mailed to a buyer (3).

One form of internet-facilitated sex work that has gained popularity across college campuses is arranged relationships, colloquially known as “sugar dating.” Websites such as *SugarDaddyMeet* and *Sugarbabies* pair college students with wealthy, older, “mentors” who finance their tuition in exchange for regular dates. Arrangement sites tout their success in providing college students, or “Sugar Babies,” with the opportunity to connect with Sugar Daddies and Sugar Mamas to help alleviate the financial burden of pursuing higher education (4). Advertising for arrangement sites is often found in social media through affiliate programs, where social media users are paid to share content (5, 6).

In 2021, over 3 million U.S. students sought financial assistance by signing up for sugar dating platforms. Seeking Arrangement was mentioned in a high-profile investigation with trafficking allegations involving a former Congressman and a minor (7, 8). After years dedicated to marketing to university students and releasing yearly top 20 university rankings, the popular *Seeking Arrangement* sugar dating site rebranded to *Seeking* in early 2022 (9) (Press@seeking.com, personal communication, March 11, 2025). *Seeking* has since partnered with non-profit organization Freedom Light to prevent human trafficking recruitment through online dating by training *Seeking* staff and monitoring dating profiles with AI and no longer condones any type of transactional dating (10).

While many sugar babies distance themselves from the term “sex worker,” (11) research on sex work in the university setting can help shed light on this phenomenon. Sagar et al. (12) examined sex work among college students in the UK to understand motivations and experiences in the sex work industry. Of the sampled enrolled students, 4.8% (*n* = 6,773) reported doing sex work in some capacity. About 57% of the students used income from sex work to pay for higher education, while 45% and 39% did so to avoid or reduce debt, respectively. Of the participants who reported doing sex work, 14% responded that they felt forced to work in the sex industry and were more likely to report fear of violence, not feeling safe during their work, and diminished self-esteem. Additionally, Sagar et al. (12) found that university faculty and staff did not have enough knowledge about students in the sex work industry to be able to provide proper referrals to students in need.

More recently, in the United States, Gerassi et al. (13) reported that 4.5% of sampled students from a large, public, Northeast university participated in exchanging sex for money or other forms of compensation. Childhood trauma, alcohol use problems, and mental health challenges before the age of 18 were associated with exchanging sex for money while at university. Similarly, sexual abuse, drug use, and housing stability were associated with trading sex in a study involving the National Longitudinal Study of Adolescent Health (14).

Stewart (15) highlighted the risk of stigmatization and stereotyping among college sex workers, especially among minorities and those with marginalized identities. While stigma is something that can affect all sex workers, racial, and sexual minorities often face added scrutiny when dealing with law enforcement and institutions like higher education. The college sex workers in Stewart's (15) study acknowledged their close proximity to violence and occasional negative experiences in their work.

Park et al. (16) noted that street-based sex workers experienced high rates of violence, contributing to Posttraumatic Stress Disorder (PTSD) symptoms and levels of PTSD similar to those of war veterans. Prevalence of lifetime violence among sex workers was 81.8%, with 61.4% meeting PTSD thresholds. Childhood and adult sexual violence experiences were significantly associated with PTSD symptoms.

Sex work among college students remains severely understudied, despite heightened risk for sexual victimization, mental health challenges, and financial stressors of this group. Therefore, the purpose of this study was to measure the prevalence of sex work among a sample of undergraduate students and examine whether engagement in sex work during college is associated with sexual victimization, symptoms of PTSD, anxiety, and depression.

## Method

### Sample and procedures

Participants in this study were included from an ongoing longitudinal cohort study of college students at a large, urban, mid-Atlantic public university (17). For a detailed review of study methods see (18). This study was approved by the university's institutional review board and all participants provided informed consent. Incoming first-year students who are at least 18 years of age were invited to complete a baseline survey their first semester of college and complete follow-up surveys every year until graduation. Survey completion was incentivized with a $15 e-gift card. In the Spring of 2024, 149 participants completed the S4S parent survey in cohort 6, and 307 in cohort 7, for a total of 455 survey respondents. The sample was predominantly female (69%), straight (58%), and POC (47%).

Data collection for this study began in Spring of 2024 and occurred during Spit for Science follow-up surveys in cohorts 6 and 7, meaning participants were in their junior and senior years of college. Study data were collected and managed using REDCap (Research Electronic Data Capture) electronic data capture tools. REDCap is a secure, web-based application designed to support data capture for research studies (19, 20).

### Measures

#### Interpersonal violence and potentially exploitive trauma experiences

A modified *Gender-Based Violence/Harassment Questionnaire (GBVHQ)* contained six questions assessing for sexual exploitation, sex work activities (such as sugar dating and camming), intimate partner violence, and sexual assault. This questionnaire was utilized in prior Campus Climate Surveys on Sexual Violence and Bystander Behavior (21). The questionnaire was developed using behavioral language, a technique widely used in campus climate surveys known to produce greater prevalence accuracy than legal terms alone, and was reviewed by content and survivor experts (21, 36). The GBVHQ distinguishes between arrangement relationships (sugar dating) and internet-facilitated sex work by asking the following: “*Since enrolling [in college], have you agreed to participate in arrangement relationships, (i.e., “sugar daddies/babies”) to help fund tuition and other financial obligations/needs?”* and “*Since enrolling [in college], have you shared images, videos, or live-streamed sexual content of yourself in exchange for money? (camming, OnlyFans, etc.)?”* These responses were dichotomized into a “yes/no” variable and combined to create one dichotomous “sex work” variable for analysis.

#### Childhood sexual victimization

A modified Adverse Childhood Experiences (ACE) scale assessed the prevalence of significant adverse childhood experiences that have been linked to negative adult outcomes and subsequent sexual victimization experiences. The four items measured the occurrence of sexual abuse experiences before age 18 and included sexual abuse by an adult, intimate partner violence, human trafficking before the age of 18, and peer sexual assault (22–24, 33). The responses to these questions were dichotomized into “yes/no” variables for analysis.

#### Posttraumatic stress disorder

The Posttraumatic Stress Disorder Checklist for DSM-5 (PCL-5) PTSD symptom severity was assessed using the PCL-5, a 20-item self-report instrument that measures the 20 DSM-5 symptoms of PTSD (25). Participants rated each item on a 5-point Likert scale ranging from 0 to 4, with total scores ranging from 0 to 80. Responses of those who answered 16 or more of the 20 items were considered for analysis. Higher scores indicate greater PTSD symptom severity, with a score of 31–33 often utilized as a clinical cutoff for probable PTSD. In the current study, the PCL-5 demonstrated excellent internal consistency (Cronbach's α = 0.966, *N* = 153).

#### Anxiety

Anxiety was measured using four items from the Symptom Checklist-90-Revised (SCL-90-R) (26). Participants rated the extent to which they were distressed by specific symptoms over the past 30 days, including: (1) nervousness or shakiness inside, (2) suddenly scared for no reason, (3) feeling fearful, and (4) spells of terror or panic. Responses were recorded on a 5-point Likert scale ranging from 1 (*Not at all*) to 5 (*Extremely*). A composite mean score for participants who completed at least 75% of the subscale items was calculated. Higher scores indicate greater levels of anxiety-related distress. In the current sample (*N* = 410), the four-item composite demonstrated strong internal consistency, with a Cronbach's α of 0.87.

#### Depression

Depression was measured using four items from the Symptom Checklist-90-Revised (SCL-90-R) (26). Participants rated the extent to which they were distressed by specific symptoms over the past 30 days, and responses were recorded on a 5-point Likert scale ranging from 1 (*Not at all*) to 5 (*Extremely*). A composite mean score for participants who completed at least 75% of the subscale items was calculated. Higher scores indicate greater levels of depression-related distress. In the current sample (*N* = 410), the four-item composite demonstrated strong internal consistency, with a Cronbach's α of 0.85.

### Data analysis

Descriptive statistics and regressions were conducted using SPSS Statistics for Windows, Version 31.0 (27). Prior to conducting the linear regression, a series of independent sample *t*-tests were performed to identify significant covariates. Only variables demonstrating a significant bivariate relationship with PTSD total scores (*p* < 0.05) were included as predictors in the final regression model.

To test the primary hypothesis, a multiple linear regression was performed to determine the contribution of sex work history on PTSD symptoms, while controlling for other significant trauma histories. Prior to the regression, assumptions of normality, homoscedasticity, and multicollinearity were assessed. All Variance Inflation Factors (VIF) were below 1.3, indicating no concerns regarding multicollinearity. The distribution of residuals showed a slight right skew in this university population, and the P-P Plot met assumptions for normality.

The larger sample size for the anxiety and depression models (*N* = 410) allowed for a hierarchical approach to examine college-era traumas above and beyond childhood history. A hierarchical linear regression was conducted to examine college-era trauma and childhood trauma on anxiety symptoms. Variables with independent associations were included in Block 1 (Childhood Peer SA, Pre 18 IPV, and Childhood Trafficking) and Block 2 (College SA, Sex Work, Human Trafficking, and College IPV). Similarly, variables with independent associations to depression symptoms were used in a hierarchical linear regression. Block 1 included Childhood Peer SA, Pre 18 IPV, Adult Perp CSA, and Childhood Trafficking, and Block 2 included Sex Work, Trafficking, and College SA.

## Results

### Preliminary analysis

As shown in Table 1, of the overall sample of 455, 6% (*n* = 27) of respondents reported having participated in arranged relationships, and 4% (*n* = 17) of students participated in live-streaming or in selling images for money. Forty-five percent (*n* = 204) reported a sexual victimization experience before the age of 18. Out of the total sample, 150 participants (33%) had at least one GBVHQ experience. The majority of those with GBVHQ experiences identified as cisgender (77%), non-white (52%), LGBQ (59%), women (93%), with an average age of 20.9 years old. Sixty eight percent (*n* = 102) of this sample reported at least one sexual victimization experience before the age of 18. Table 1 presents GBVHQ percentages within each demographic category. While females and cisgender women faced higher percentages of GBVHQ in the sex assigned at birth and gender identity categories, LGBQ participants reported a higher frequency of participation in sex work (arrangement relationships and internet facilitated sex work) and experienced a higher percentage of IPV and sexual assault compared to heterosexual participants. More POC (Asian, Black, or Hispanic) participants reported participation in sex work and experiencing sex trafficking than White or Multiracial participants.

**Table 1:** GBHQ by demographics.

Characteristic		Arrangement	Trafficking	Internet Sex Work	IPV	SA
		*n* = (27) 6%	*n* = (25) 6%	*n* = (17) 4%	*n* = (50) 12%	*n* = (87) 20%
Sex	Female	(24) 89%	(22) 88%	(15) 94%	(48) 98%	(80) 92
	Male	(3) 11%	(3) 12%	(1) 6%	(1) 2%	(7) 8%
Gender Id	Ciswomen	(17) 63%	(21) 84%	(11) 65%	(40) 80%	(69) 79%
	Cismen	(3) 11 %	(1) 4%	(1) 6%	(1) 2%	(4) 5%
	Trans & Non-binary	(8) 29%	(3) 12%	(5) 30%	(9) 18%	(15) 18%
SO	Heterosexual	(12) 44%	(12) 48%	(8) 47%	(20) 40%	(30) 35%
	LGBQ	(15) 56%	(13) 52%	(9) 53%	(30) 60%	(57) 66%
Race	POC	(13) 38%	(11) 44%	(6) 35%	(15) 30%	(30) 35%
	Multiracial	(4) 15%	(4) 16%	(4) 24%	(12) 24%	(13) 15%
	White	(8) 30%	(8) 32%	(6) 35%	(22) 44%	(41) 47%

*N* = 150.

The full sample mean PCL-5 scores (*M* = 23.9) fell below the clinical threshold for PTSD, while those who participated in sex work reported a mean score of 31.1, which is within the commonly used clinical threshold of 31–33. Initial analyses revealed several significant predictors of PTSD symptoms. Independent sample *t*-tests showed that participants with a history of sex work (*M* = 31.1) reported significantly higher PCL-5 scores than those without (*M* = 21.8), *t*_(149)_ = −1.71, *p* = 0.045 (one-tailed). The effect size was Cohen's *d* = 0.41, indicating a medium effect.

Similarly, significant differences in PCL-5 scores were found for those with histories of college-based sexual assault, IPV during college, and childhood trafficking (*p* < 0.05). Based on these results, these four variables were entered into the linear regression model.

A Chi-square test of independence was performed to examine the relationship between sex work history and college-based sexual assault. The relationship was significant, χ^2^(1) = 20.65, *p* < 0.001. Participants with a history of sex work were significantly more likely to report experiencing sexual assault during college (50.0%) compared to participants without (17.5%).

### Primary analysis

A multiple linear regression was conducted to predict total PCL-5 scores based on sex work history and co-occurring trauma variables. The overall regression model was statistically significant, *F*_(4, 146)_ = 3.36, *p* = 0.012, indicating that the predictors collectively accounted for 9% of the variance in PTSD symptoms (see Table 2).

**Table 2:** Multiple linear regression model PCL-5.

Predictor	*B*	SE	β	*t*	*p*
(Constant)	18	2.18	–	8.27	< 0.001
Child Trafficking	5.72	4.14	0.116	1.38	0.169
**College SA**	**8.37**	**3.52**	**0.207**	**2.38**	**0.019**
Sex Work	1.75	5.31	0.03	0.33	0.742
College IPV	4.49	4.32	0.094	1.04	0.301

R^2^ = 0.09; Adjusted R^2^ = 0.07; F_(4, 146)_ = 3.36, p = 0.012.

In this model, college-based sexual assault was the sole significant predictor of PCL-5 scores (*B* = 8.37, β = 0.207, *p* = 0.019). While sex work history was associated with higher scores in initial bivariate comparisons, it did not reach statistical significance in the multivariate model (*p* = 0.742). Similarly, IPV during college and childhood trafficking were not unique significant predictors when accounting for college-based sexual assault.

Sex work was independently associated with higher anxiety symptoms in bivariate analyses, *t*_(407)_ = 3.24, *p* = 0.001, with a mean score of 2.53 (out of 5). In the final multivariate anxiety model, childhood peer SA (β = 0.15, *p* = 0.013) emerged as the only significant independent predictors of anxiety (see Table 3). College SA (β = 0.12, *p* = 0.051) and Sex Work (β = 0.10, *p* = 0.066) approached significance, suggesting that more recent experiences may be associated with increased anxiety distress; however, their individual effects were attenuated when accounting for childhood history. The overall model was statistically significant, *F*_(7, 363)_ = 4.48, *p* < 0.001. Block 1, which included childhood sexual trauma variables, accounted for 4% of the variance in anxiety. The addition of college-era traumas in Block 2 accounted for an 8% of the variance, *F* change = 4.32, *p* = 0.002.

**Table 3:** Hierarchical linear regression model.

Predictor	Anxiety (β)	Depression (β)
Block 1: Childhood		
Childhood SA	0.12^*^	0.15^*^
Child trafficking	0.004	0.02
IPV < 18	0.06	0.07
Adult Perp CSA	—	0.04
Block 2: College		
College SA	0.12	0.13^*^
Sex work	0.1	0.11^**^
Trafficking	0.08	0.06
College IPV	0.05	—
—	—	—
R^2^	0.04^**^	0.05^**^
ΔR^2^	0.08^***^	0.08^***^

^*^p < 0.05;

^**^p < 0.01;

^***^p < 0.001.

Sex work was independently associated with higher depression symptoms in bivariate analyses, *t*_(407)_ = 3.56, *p* = 0.001, with a mean score of 3.24 (out of 5). In the hierarchical linear regression model, after controlling for childhood trauma and other college-era violence, involvement in sex work in college remained a significant predictor of higher levels of depression (*p* = 0.042). Additionally, College SA (β = 0.13, *p* = 0.032) and Childhood Peer SA (β = 0.14, *p* = 0.021) emerged as significant independent predictors of depression symptoms. The overall model was statistically significant *F*_(7, 359)_ = 4.23, *p* < 0.001 (see Table 3). Block 1, which included childhood sexual trauma variables, accounted for 3.4% of the variance in depression symptoms. The addition of college-era trauma variables in Block 2 accounted for an additional 8.1% of the variance, *F* change = 4.79, *p* = 0.003.1

## Discussion

### Limitations

This study has several limitations. The PCL-5 scale had a low response rate of 33%. The reason for this high attrition rate is unknown, though it may be due to the length of the scale or nature of the questions related to trauma. Additionally, this scale was calculated to count responses from participants who answered 16 or more of the 20 questions to prevent skewing. Comparatively, the short anxiety and depression scales had much higher response rates, at 93% and 90%, respectively, even while only counting responses for participants who completed 75% of the scales. Further, the *t*-test with sex work and PCL-5 scale variables was significant (*p* = 0.45) while accounting for a directional hypothesis, however, this result would not be significant with a two-tailed *t*-test. While causality of PTSD, anxiety, and depression cannot be inferred from cross-sectional analyses, they are appropriate for identifying associations, particularly when examining relationships grounded in well-established theoretical and diagnostic frameworks, such as the link between adverse childhood experiences and PTSD symptom severity and depression in young adulthood (28).

### Implications

This study contributes to the growing literature on sexual violence and college student mental health by examining sex work as a contextual factor associated with trauma exposure, victimization risk, and psychological distress among college students. Consistent with prior research documenting the recurrent nature of violence across the lifespan (CDC, 2024), and specifically during college (21, 29), our findings suggest that students engaged in sex work during college reported significantly higher levels of anxiety and depression symptoms, as well as higher rates of sexual assault, compared to their peers. These associates persisted even after accounting for childhood sexual trauma and other college-era victimization, highlighting the importance of examining sex work within a broader framework of cumulative risk and structural vulnerability.

From a public health perspective, it is important to consider these results not as inherent to sex work itself, but rather as reflective of the broader social, economic, and environmental contexts in which college sex work occurs. Financial stress (30), housing insecurity (31), and stigma related to both sex work and mental health service utilization (35) may increase students' exposure to high risk situations and decrease their ability to access protective resources. It is important to consider how these contexts overlap with interlocking systems of oppression such as gender, race, class, and sexuality, which may amplify vulnerability.

Our findings suggest that structural vulnerabilities tied to identity characteristics may increase both engagement in sex work and risk for sexual victimization. One-third of participants experienced at least one victimization or potentially exploitative event in college, with the majority identifying as cisgender women (77%), non-white (52%), and LGBQ (59%), and over two-thirds of these students reported sexual victimization prior to age 18. Sex work was reported more frequently among LGBQ students and students of color. These intersecting vulnerabilities rooted in structural inequality may increase risk for sexual victimization and exacerbate mental health symptoms through stress proliferation and cumulative trauma exposure, and contribute to disproportionate health risks among historically underserved student populations. Our findings align with prior research that established that sex workers experience elevated rates of violence across the lifespan, contributing to mental health issues (16). Racially and sexually minoritized sex workers may be at increased risk for violence and adverse mental health outcomes due to fetishization and objectification by clients compounded with the burden of secrecy due to social stigma (15).

Importantly, while sex work is a consensual form of labor, it can occur within hierarchical and power-imbalanced contexts that increase exposure to coercion or assault. These contexts are inherent within the college environment, with embedded academic (e.g., student, TA, advisor, professor) and social (e.g., seniority within athletics, Greek life, and other social organizations) power imbalances. It is critical to distinguish consensual labor from sexual victimization to avoid stigmatizing sex-working students, while also acknowledging the heightened risk environments they may inhabit. The finding that students engaged in sex work experienced higher rates or sexual assault than their peers likely reflects contextual and environmental risk rather than sex work itself, reinforcing the need for nuanced, sex positive public health approaches.

Our findings also extend existing literature on revictimization by showing that childhood sexual abuse, including peer sexual abuse, is a significant predictor of anxiety and depression in college. Collectively, these results indicate that college sex workers carry an increased burden of PTSD, anxiety, and depression symptoms compared to their peers, alongside higher reported rates of sexual assault.

Given the unique role of the college environment in student wellbeing, these findings have important implications for campus policy and practice. Universities are well-positioned to provide students resources like Title IX support, health care services, and counseling. Student facing providers should be aware of the presence of sex workers on campus and be prepared to assess and provide resources for sexual violence, housing insecurity, financial stress, and mental health needs. Future prevention and response interventions and campus policies should consider integrating trauma-informed, sex-positive approaches that address both mental health and structural vulnerabilities.

In addition to identifying risk factors, it is important to integrate protective factors that may mediate mental health concerns and victimization risk among college students engaged in sex work. Research on sex work more broadly, and sexual victimization among college students, suggest that social support (34) and social cohesion (32) are associated with lower odds of physical and sexual violence, revictimization, buffer against harm. Fostering supportive peer communities, accessible trauma-informed counseling, and educating students on harm-reduction practices when engaging in sex work are important areas for future research.

## Data Availability

Data from this study are available to qualified researchers via dbGaP (phs001754.v4.p2) or via spit4science@vcu.edu to qualified researchers who provide the appropriate signed data use agreement.

## Group members of Spit for Science Work Group

The Spit for Science Working Group: Director: Karen Chartier. Co-Director: Ananda Amstadter. Past Founding Director: Danielle M. Dick (2011-2022). Registry management: Emily Lilley, Renolda Gelzinis, Anne Morris. Data cleaning and management: Katie Bountress, Amy E. Adkins, Nathaniel Thomas, Zoe Neale, Kimberly Pedersen, Thomas Bannard & Seung B. Cho. Data collection: Kimberly Pedersen, Amy E. Adkins, Peter Barr, Holly Byers, Erin C. Berenz, Erin Caraway, Seung B. Cho, James S. Clifford, Megan Cooke, Elizabeth Do, Alexis C. Edwards, Neeru Goyal, Laura M. Hack, Lisa J. Halberstadt, Sage Hawn, Sally Kuo, Emily Lasko, Jennifer Lent, Mackenzie Lind, Elizabeth Long, Alexandra Martelli, Jacquelyn L. Meyers, Kerry Mitchell, Ashlee Moore, Arden Moscati, Aashir Nasim, Zoe Neale, Jill Opalesky, Cassie Overstreet, A. Christian Pais, Tarah Raldiris, Jessica Salvatore, Jeanne Savage, Rebecca Smith, David Sosnowski, Jinni Su, Nathaniel Thomas, Chloe Walker, Marcie Walsh, Teresa Willoughby, Madison Woodroof & Jia Yan.
